# 2-Chloro-*N*-isopropyl-*N*-phenyl­acetamide

**DOI:** 10.1107/S1600536810027157

**Published:** 2010-07-14

**Authors:** Mao-Sen Yuan, Zhi-peng Li, Qi Wang

**Affiliations:** a College of Science, Northwest A&F University, Yangling 712100, Shannxi Province, People’s Republic of China; b College of Life Sciences, Northwest A&F University, Yangling 712100, Shannxi Province, People’s Republic of China

## Abstract

In the title compound, C_11_H_14_ClNO, the herbicide propachlor, there are significant differences between the three N—C bond lengths [N—C_carbon­yl_ = 1.354 (3) Å, N—C_phen­yl_ = 1.444 (2) Å and N—C_isoprop­yl_ = 1.496 (3) Å], indicating the presence of π delocalization involving the carbonyl group. The N atom lies 0.074 (2) Å from the plane defined by the the three bonded C atoms.

## Related literature

For studies of propachlor and its derivatives, see: Dhillon & Anderson (1972 ▶); Kleudgen (1980 ▶).
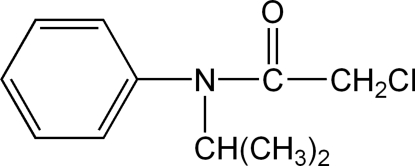


## Experimental

### 

#### Crystal data

C_11_H_14_ClNO
*M* *_r_* = 211.68
Monoclinic, 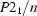

*a* = 11.9190 (11) Å
*b* = 7.8042 (8) Å
*c* = 12.3789 (13) Å
β = 98.963 (1)°
*V* = 1137.4 (2) Å^3^
*Z* = 4
Mo *K*α radiation
μ = 0.30 mm^−1^
*T* = 298 K
0.47 × 0.45 × 0.44 mm

#### Data collection

Bruker APEXII CCD diffractometer
Absorption correction: multi-scan (*SADABS*; Bruker, 2005 ▶) *T*_min_ = 0.870, *T*_max_ = 0.878
5426 measured reflections
2004 independent reflections
1501 reflections with *I* > 2σ(*I*)
*R*_int_ = 0.025

#### Refinement

*R*[*F* ^2^ > 2σ(*F* ^2^)] = 0.038
*wR*(*F* ^2^) = 0.111
*S* = 1.03
2004 reflections
130 parameters
H-atom parameters constrained
Δρ_max_ = 0.21 e Å^−3^
Δρ_min_ = −0.19 e Å^−3^

### 

Data collection: *SMART* (Bruker, 1997 ▶); cell refinement: *SAINT* (Bruker, 1997 ▶); data reduction: *SAINT*; program(s) used to solve structure: *SHELXS97* (Sheldrick, 2008 ▶); program(s) used to refine structure: *SHELXL97* (Sheldrick, 2008 ▶); molecular graphics: *SHELXTL* (Sheldrick, 2008 ▶); software used to prepare material for publication: *SHELXTL*.

## Supplementary Material

Crystal structure: contains datablocks global, I. DOI: 10.1107/S1600536810027157/zs2048sup1.cif

Structure factors: contains datablocks I. DOI: 10.1107/S1600536810027157/zs2048Isup2.hkl

Additional supplementary materials:  crystallographic information; 3D view; checkCIF report

## Figures and Tables

**Fig. 1.**
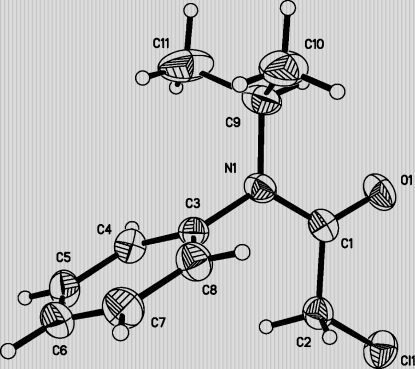
The molecular comformation and atom numbering scheme for (I). Displacement ellipsoids are drawn at the 30% probability level.

**Fig. 2.**
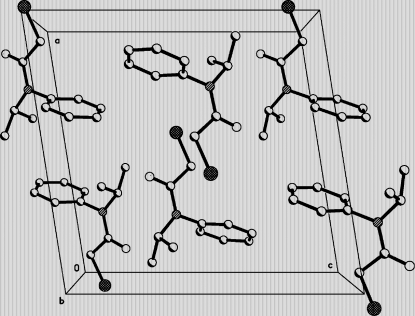
The molecular packing of (I) viewed down the b axis of the unit cell.

## supplementary crystallographic information

### Comment

Propachlor (2-chloro-*N*-isopropyl-*N*-phenylacetamide) and its derivatives have been widely studied as a pre-emergent herbicide used to control broadleaf weeds and grasses (Dhillon *et al.*, 1972; Kleudgen, 1980). Propachlor may also be used as a precursor in the synthesis of indole-2-one compounds and in the course of exploring new indole-2-one compounds, we synthesized the title compound C_11_H_14_ClNO (I), the structure of which is reported here.

In structure of (I) (Fig. 1), there are obvious differences between the three C—N bond lengths (N—C_carbonyl_, 1.354 (3) Å; N—C_phenyl_, 1.444 (2) Å; N—C_isopropyl_, 1.496 (3) Å, indicating the presence of π delocalization involving the carbonyl group. Also N1 lies close to the plane defined by the three bonded carbon atoms C1, C3 and C9  [0.074 (2) Å].

As expected, there are no classic hydrogen bonds in the structure (Fig. 2). However, there is a weak intermolecular aliphatic C11—H11A···O1^i^ interaction [symmetry code: (i) -*x* + 1/2, *y* - 1/2, -*z* + 1/2] stabilizing the packing. This intermolecular hydrogen bond is characterized by the parameters 0.96 Å (C11—H11A) and 2.56 Å (H11A···O1^i^).

### Experimental

*N*-Isopropylbenzenamine (1.00 g, 7.41 mmol) was dissolved in toluene (5.0 mL) and cooled to 273 K, after which a solution of 2-chloroacetyl chloride (0.90 g, 8.03 mmol) in toluene (5.0 mL) was slowly added over 0.5 h. with stirring. The mixture was then refluxed for 2 h. then slowly cooled to room temperature over 8 h. Colorless block crystals of (I) were formed (1.33 g, yield 85%).

### Refinement

All H atoms were placed in geometrically calculated positions and refined using a riding model with C—H_aromatic_ = 0.93 %A and C—H_aliphatic_ = 0.96–0.97 %A, with *U*_iso_ = 1.2*U*_eq_(C), or 1.5*U*_eq_(C) for CH_3_ groups.

### Crystal data

**Table:**

C_11_H_14_ClNO	*F*(000) = 448
*M**_r_* = 211.68	*D*_x_ = 1.236 Mg m^−^^3^
Monoclinic, *P*2_1_/*n*	Mo *K*α radiation, λ = 0.71073 Å
Hall symbol: -P 2yn	Cell parameters from 2899 reflections
*a* = 11.9190 (11) Å	θ = 2.2–25.0°
*b* = 7.8042 (8) Å	µ = 0.30 mm^−^^1^
*c* = 12.3789 (13) Å	*T* = 298 K
β = 98.963 (1)°	Block, colorless
*V* = 1137.4 (2) Å^3^	0.47 × 0.45 × 0.44 mm
*Z* = 4	

### Data collection

**Table:**

Bruker APEXII CCD diffractometer	2004 independent reflections
Radiation source: fine-focus sealed tube	1501 reflections with *I* > 2σ(*I*)
graphite	*R*_int_ = 0.025
φ and ω scans	θ_max_ = 25.0°, θ_min_ = 2.2°
Absorption correction: multi-scan (*SADABS*; Bruker, 2005)	*h* = −14→12
*T*_min_ = 0.870, *T*_max_ = 0.878	*k* = −9→8
5426 measured reflections	*l* = −14→14

### Refinement

**Table:**

Refinement on *F*^2^	Secondary atom site location: difference Fourier map
Least-squares matrix: full	Hydrogen site location: inferred from neighbouring sites
*R*[*F*^2^ > 2σ(*F*^2^)] = 0.038	H-atom parameters constrained
*wR*(*F*^2^) = 0.111	*w* = 1/[σ^2^(*F*_o_^2^) + (0.0469*P*)^2^ + 0.4408*P*] where *P* = (*F*_o_^2^ + 2*F*_c_^2^)/3
*S* = 1.03	(Δ/σ)_max_ < 0.001
2004 reflections	Δρ_max_ = 0.21 e Å^−^^3^
130 parameters	Δρ_min_ = −0.19 e Å^−^^3^
0 restraints	Extinction correction: *SHELXL97* (Sheldrick, 2008), Fc^*^=kFc[1+0.001xFc^2^λ^3^/sin(2θ)]^-1/4^
Primary atom site location: structure-invariant direct methods	Extinction coefficient: 0.079 (5)

### Special details

**Table:**

Geometry. All e.s.d.'s (except the e.s.d. in the dihedral angle between two l.s. planes) are estimated using the full covariance matrix. The cell e.s.d.'s are taken into account individually in the estimation of e.s.d.'s in distances, angles and torsion angles; correlations between e.s.d.'s in cell parameters are only used when they are defined by crystal symmetry. An approximate (isotropic) treatment of cell e.s.d.'s is used for estimating e.s.d.'s involving l.s. planes.
Refinement. Refinement of *F*^2^ against ALL reflections. The weighted *R*-factor *wR* and goodness of fit *S* are based on *F*^2^, conventional *R*-factors *R* are based on *F*, with *F* set to zero for negative *F*^2^. The threshold expression of *F*^2^ > σ(*F*^2^) is used only for calculating *R*-factors(gt) *etc*. and is not relevant to the choice of reflections for refinement. *R*-factors based on *F*^2^ are statistically about twice as large as those based on *F*, and *R*- factors based on ALL data will be even larger.

### Fractional atomic coordinates and isotropic or equivalent isotropic displacement parameters (Å^2^)

**Table:**

	*x*	*y*	*z*	*U*_iso_*/*U*_eq_	
Cl1	0.57852 (5)	0.22476 (9)	0.44873 (5)	0.0711 (3)	
N1	0.25393 (14)	0.3526 (2)	0.40187 (13)	0.0483 (4)	
O1	0.40481 (14)	0.4455 (2)	0.32694 (12)	0.0662 (5)	
C1	0.36637 (17)	0.3629 (3)	0.39670 (16)	0.0475 (5)	
C2	0.44299 (18)	0.2648 (3)	0.48500 (18)	0.0543 (6)	
H2A	0.4074	0.1566	0.4980	0.065*	
H2B	0.4520	0.3301	0.5525	0.065*	
C3	0.21346 (16)	0.2664 (3)	0.49159 (15)	0.0438 (5)	
C4	0.17401 (18)	0.0998 (3)	0.47994 (18)	0.0548 (6)	
H4	0.1734	0.0423	0.4140	0.066*	
C5	0.1354 (2)	0.0186 (3)	0.5666 (2)	0.0674 (7)	
H5	0.1097	−0.0939	0.5592	0.081*	
C6	0.1349 (2)	0.1042 (4)	0.6635 (2)	0.0691 (7)	
H6	0.1073	0.0504	0.7211	0.083*	
C7	0.1751 (2)	0.2691 (4)	0.67528 (19)	0.0701 (7)	
H7	0.1757	0.3260	0.7414	0.084*	
C8	0.21471 (19)	0.3513 (3)	0.58983 (17)	0.0586 (6)	
H8	0.2420	0.4629	0.5983	0.070*	
C9	0.1714 (2)	0.4559 (3)	0.32459 (18)	0.0641 (7)	
H9	0.2083	0.4850	0.2615	0.077*	
C10	0.1415 (3)	0.6210 (3)	0.3759 (3)	0.0939 (10)	
H10A	0.1033	0.5964	0.4368	0.141*	
H10B	0.0926	0.6875	0.3228	0.141*	
H10C	0.2097	0.6845	0.4006	0.141*	
C11	0.0654 (3)	0.3538 (4)	0.2827 (3)	0.1013 (11)	
H11A	0.0865	0.2471	0.2530	0.152*	
H11B	0.0186	0.4179	0.2267	0.152*	
H11C	0.0240	0.3318	0.3418	0.152*	

### Atomic displacement parameters (Å^2^)

**Table:**

	*U*^11^	*U*^22^	*U*^33^	*U*^12^	*U*^13^	*U*^23^
Cl1	0.0516 (4)	0.0819 (5)	0.0834 (5)	0.0023 (3)	0.0212 (3)	−0.0015 (3)
N1	0.0526 (10)	0.0511 (10)	0.0426 (9)	0.0058 (8)	0.0115 (7)	0.0076 (8)
O1	0.0760 (11)	0.0647 (11)	0.0635 (9)	−0.0032 (8)	0.0288 (8)	0.0168 (8)
C1	0.0592 (13)	0.0403 (11)	0.0455 (11)	−0.0013 (9)	0.0157 (10)	−0.0021 (9)
C2	0.0477 (12)	0.0591 (14)	0.0588 (13)	0.0012 (10)	0.0167 (10)	0.0089 (11)
C3	0.0410 (10)	0.0493 (12)	0.0420 (10)	0.0039 (9)	0.0091 (8)	0.0049 (9)
C4	0.0519 (12)	0.0549 (14)	0.0582 (13)	−0.0021 (10)	0.0106 (10)	−0.0024 (11)
C5	0.0572 (14)	0.0606 (15)	0.0859 (17)	−0.0076 (11)	0.0157 (12)	0.0164 (14)
C6	0.0581 (14)	0.090 (2)	0.0622 (15)	−0.0004 (13)	0.0178 (11)	0.0268 (15)
C7	0.0761 (17)	0.091 (2)	0.0456 (13)	−0.0016 (14)	0.0178 (12)	0.0005 (13)
C8	0.0685 (14)	0.0576 (14)	0.0514 (12)	−0.0041 (11)	0.0150 (10)	−0.0015 (11)
C9	0.0692 (15)	0.0715 (17)	0.0521 (12)	0.0177 (12)	0.0110 (11)	0.0201 (12)
C10	0.095 (2)	0.0512 (16)	0.122 (2)	0.0114 (15)	−0.0266 (18)	0.0041 (16)
C11	0.112 (2)	0.078 (2)	0.094 (2)	0.0226 (18)	−0.0480 (18)	−0.0207 (17)

### Geometric parameters (Å, °)

**Table:**

Cl1—C2	1.771 (2)	C6—C7	1.374 (4)
N1—C1	1.354 (3)	C6—H6	0.9300
N1—C3	1.444 (2)	C7—C8	1.381 (3)
N1—C9	1.496 (3)	C7—H7	0.9300
O1—C1	1.222 (2)	C8—H8	0.9300
C1—C2	1.518 (3)	C9—C10	1.503 (4)
C2—H2A	0.9700	C9—C11	1.515 (4)
C2—H2B	0.9700	C9—H9	0.9800
C3—C4	1.383 (3)	C10—H10A	0.9600
C3—C8	1.383 (3)	C10—H10B	0.9600
C4—C5	1.385 (3)	C10—H10C	0.9600
C4—H4	0.9300	C11—H11A	0.9600
C5—C6	1.373 (4)	C11—H11B	0.9600
C5—H5	0.9300	C11—H11C	0.9600
			
C1—N1—C3	121.03 (16)	C6—C7—C8	120.6 (2)
C1—N1—C9	119.66 (17)	C6—C7—H7	119.7
C3—N1—C9	118.53 (16)	C8—C7—H7	119.7
O1—C1—N1	123.2 (2)	C7—C8—C3	119.5 (2)
O1—C1—C2	121.67 (19)	C7—C8—H8	120.2
N1—C1—C2	115.15 (16)	C3—C8—H8	120.2
C1—C2—Cl1	112.14 (14)	N1—C9—C10	111.53 (19)
C1—C2—H2A	109.2	N1—C9—C11	111.5 (2)
Cl1—C2—H2A	109.2	C10—C9—C11	110.8 (2)
C1—C2—H2B	109.2	N1—C9—H9	107.6
Cl1—C2—H2B	109.2	C10—C9—H9	107.6
H2A—C2—H2B	107.9	C11—C9—H9	107.6
C4—C3—C8	119.95 (19)	C9—C10—H10A	109.5
C4—C3—N1	120.46 (18)	C9—C10—H10B	109.5
C8—C3—N1	119.59 (19)	H10A—C10—H10B	109.5
C3—C4—C5	119.9 (2)	C9—C10—H10C	109.5
C3—C4—H4	120.1	H10A—C10—H10C	109.5
C5—C4—H4	120.1	H10B—C10—H10C	109.5
C6—C5—C4	120.1 (2)	C9—C11—H11A	109.5
C6—C5—H5	119.9	C9—C11—H11B	109.5
C4—C5—H5	119.9	H11A—C11—H11B	109.5
C5—C6—C7	120.0 (2)	C9—C11—H11C	109.5
C5—C6—H6	120.0	H11A—C11—H11C	109.5
C7—C6—H6	120.0	H11B—C11—H11C	109.5
			
C3—N1—C1—O1	−174.27 (19)	N1—C3—C4—C5	−179.95 (19)
C9—N1—C1—O1	−4.6 (3)	C3—C4—C5—C6	0.8 (3)
C3—N1—C1—C2	5.3 (3)	C4—C5—C6—C7	−1.5 (4)
C9—N1—C1—C2	174.98 (19)	C5—C6—C7—C8	1.0 (4)
O1—C1—C2—Cl1	−20.0 (3)	C6—C7—C8—C3	0.1 (4)
N1—C1—C2—Cl1	160.48 (15)	C4—C3—C8—C7	−0.8 (3)
C1—N1—C3—C4	−98.8 (2)	N1—C3—C8—C7	179.5 (2)
C9—N1—C3—C4	91.4 (2)	C1—N1—C9—C10	−96.3 (2)
C1—N1—C3—C8	81.0 (3)	C3—N1—C9—C10	73.7 (3)
C9—N1—C3—C8	−88.8 (2)	C1—N1—C9—C11	139.2 (2)
C8—C3—C4—C5	0.3 (3)	C3—N1—C9—C11	−50.8 (3)
